# The Effects of Hsp90α1 Mutations on Myosin Thick Filament Organization

**DOI:** 10.1371/journal.pone.0142573

**Published:** 2015-11-12

**Authors:** Qiuxia He, Kechun Liu, Zhenjun Tian, Shao Jun Du

**Affiliations:** 1 Institute of Marine and Environmental Technology, Department of Biochemistry and Molecular Biology, University of Maryland School of Medicine, Baltimore, Maryland, 21202, United States of America; 2 Biology Institute of Shandong Academy of Sciences, Jinan, Shandong, 250014, P. R. China; 3 Institute of Sports and Exercise Biology, Shaanxi Normal University, Xi’an, Shaanxi, 710062, P. R. China; University of Geneva, SWITZERLAND

## Abstract

Heat shock protein 90α plays a key role in myosin folding and thick filament assembly in muscle cells. To assess the structure and function of Hsp90α and its potential regulation by post-translational modification, we developed a combined knockdown and rescue assay in zebrafish embryos to systematically analyze the effects of various mutations on Hsp90α function in myosin thick filament organization. DNA constructs expressing the Hsp90α1 mutants with altered putative ATP binding, phosphorylation, acetylation or methylation sites were co-injected with Hsp90α1 specific morpholino into zebrafish embryos. Myosin thick filament organization was analyzed in skeletal muscles of the injected embryos by immunostaining. The results showed that mutating the conserved D90 residue in the Hsp90α1 ATP binding domain abolished its function in thick filament organization. In addition, phosphorylation mimicking mutations of T33D, T33E and T87E compromised Hsp90α1 function in myosin thick filament organization. Similarly, K287Q acetylation mimicking mutation repressed Hsp90α1 function in myosin thick filament organization. In contrast, K206R and K608R hypomethylation mimicking mutations had not effect on Hsp90α1 function in thick filament organization. Given that T33 and T87 are highly conserved residues involved post-translational modification (PTM) in yeast, mouse and human Hsp90 proteins, data from this study could indicate that Hsp90α1 function in myosin thick filament organization is potentially regulated by PTMs involving phosphorylation and acetylation.

## Introduction

Muscle fibers are composed of myofibrils, one of the most complex and highly ordered macromolecular assemblies known. Each myofibril is made up of highly organized repetitive structures called sarcomeres, the basic contractile unit in skeletal and cardiac muscles. Recent studies demonstrate that Hsp90α plays an essential role in myosin folding and sarcomere assembly [1–4]. Loss of Hsp90α1 function in zebrafish embryos results in increased myosin protein degradation and sarcomere disorganization in skeletal muscles [3, 5]. *In vitro* studies indicate that Hsp90 forms a complex with newly synthesized myosin protein and is directly involved in myosin folding and assembly [6].

Hsp90 is a highly abundant ATPase dependent molecular chaperone required for the maturation, activation, maintenance or degradation of many proteins that are referred to as ‘client’ proteins. Hsp90 is more selective than other promiscuous general chaperones [7]. The molecular mechanism underlying the client specificity is not clear. Structural analysis revealed that Hsp90 contains three structural domains, the N-terminal ATP binding domain, the middle domain involved in client protein interaction, and the C-terminal dimerization domain [8]. The ATPase activity is essential for Hsp90 function in regulating myosin thick filament formation and skeletal muscle myofibrillogenesis [3].

Recent studies indicate that post-translational modification (PTM) regulates client protein specificity and ATPase activity of molecular chaperones, such as Hsp90 [9–11]. Large numbers of PTMs have been identified in Hsp90, including phosphorylation, acetylation, S-nitrosylation, methylation, and ubiquitination [9, 11]. It has been shown that phosphorylation of Y313 in Hsp90 promotes recruitment of Aha1, a Hsp90 co-chaperone required for ATPase activation and chaperone function [12, 13]. On the other hand, acetylation of K294 in the middle domain of yeast Hsp90 regulates client protein interaction [14]. Given the diverse array of PTM in Hsp90, a theory of chaperone code has been proposed that suggests that the combinatorial array of PTMs regulates the activity of molecular chaperones, thereby orchestrating the functional organization of the proteome [9–11, 15]. However, the regulatory role of PTM on Hsp90α1 function in muscle cells is not known.

To assess the potential regulation of Hsp90α1 function by PTMs in myosin thick filament organization, we performed a knockdown and rescue assay in zebrafish embryos to systematically analyze the effects of various Hsp90α1 mutations at the conserved phosphorylation, acetylation or methylation sites on Hsp90α1 function *in vivo*. We report here that D90 in the ATP binding domain is critical for Hsp90α1 function in myosin thick filament organization. D90A mutation abolished Hsp90α1 function in thick filament organization. In addition, phosphormimetic mutations at T33 or T87 residues blocked zebrafish Hsp90α1 function in myosin thick filament organization. Similarly, K287Q acetylation mimicking mutantion repressed Hsp90α1 function in myofibril organization. In contrast, K206R and K608R hypomethylation mimicking mutations had no effect on Hsp90α1 function in thick filament organization. Collectively, these studies indicate that Hsp90α1 function in myosin thick filament organization could be regulated by potential PTMs involving phosphorylation and acetylation.

## Results

### 1. D90 in the ATP binding domain is critical for Hsp90α1 function in myosin thick filament organization

The intrinsic ATPase activity of Hsp90 is essential for chaperone cycling [16]. It has been shown that the conserved D93, G97 and T184 residues in the ATPase domain of human Hsp90 or D79, G83 and T171 residues in yeast Hsp90 are directly involved in interaction with the adenine base of ATP molecule [17, 18]. D79N mutation in yeast Hsp90 abolishes both ATP binding and hydrolysis activity [19, 20]. Consistent with these studies, a missense mutation of G94A in zebrafish Hsp90α1 (G97 equivalent residue in human Hsp90α) abolished its ATPase activity *in vitro* and biological function in myosin thick filament organization *in vivo* [3]. The role of D93 and T184 in Hsp90α1 function in muscle cells is unknown although they are highly conserved residues in the ATPase domain of Hsp90 during evolution.

**Fig 1:**
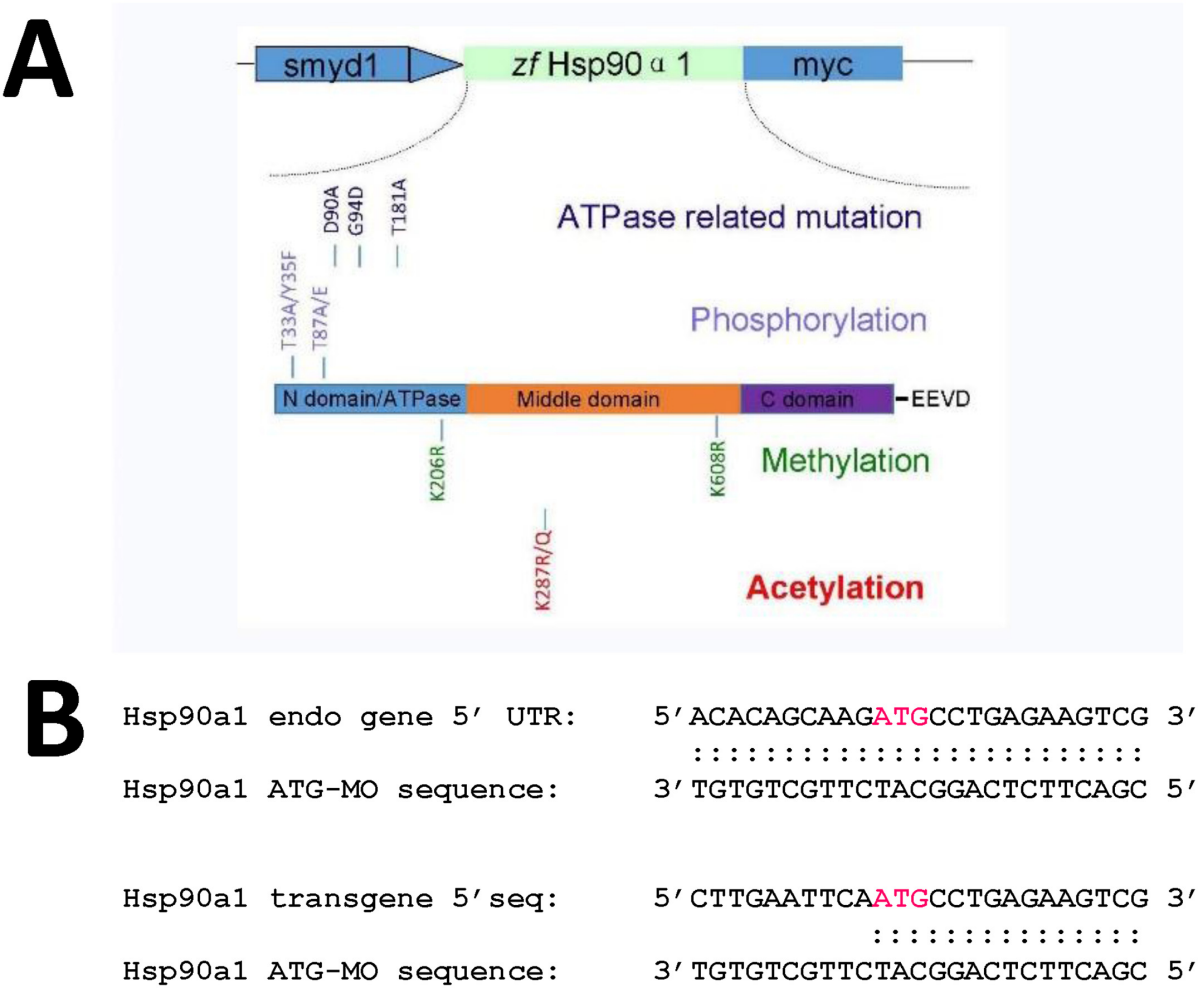
The schematic structure of various Hsp90α1 mutant constructs, and the sequence comparison of Hsp90α1 ATG-MO target in the endogenous Hsp90α1 gene and the Hsp90α1 transgenes. **A.** The schematic structure of a zebrafish Hsp90α1 transgene expressing a myc-tagged Hsp90α1 under the control of the muscle specific *smyd1* promoter. The Hsp90α1 contains 3 conserved functional domains. The N-terminal ATP binding and activation domain; the middle domain involved in cochaperone and client protein binding, and the C-terminal dimerization domain. Single and double mutations were made at the amino acid residues involved in ATP binding (D90, G94, T181), post-translational modification by phosphorylation (T33, Y35, T87), acetylation (K287) or methylation (K206, K608). The amino acid substitutions and their positions are indicated. **B.** DNA sequence comparison of Hsp90α1 ATG-MO target in the endogenous gene and the Hsp90α1 transgene. Half of the ATG-MO target sequence in the Hsp90α1 transgene has been replaced with an EcoRI site and part of the myc-tag sequence from the CS2-MT vector.

**Fig 2:**
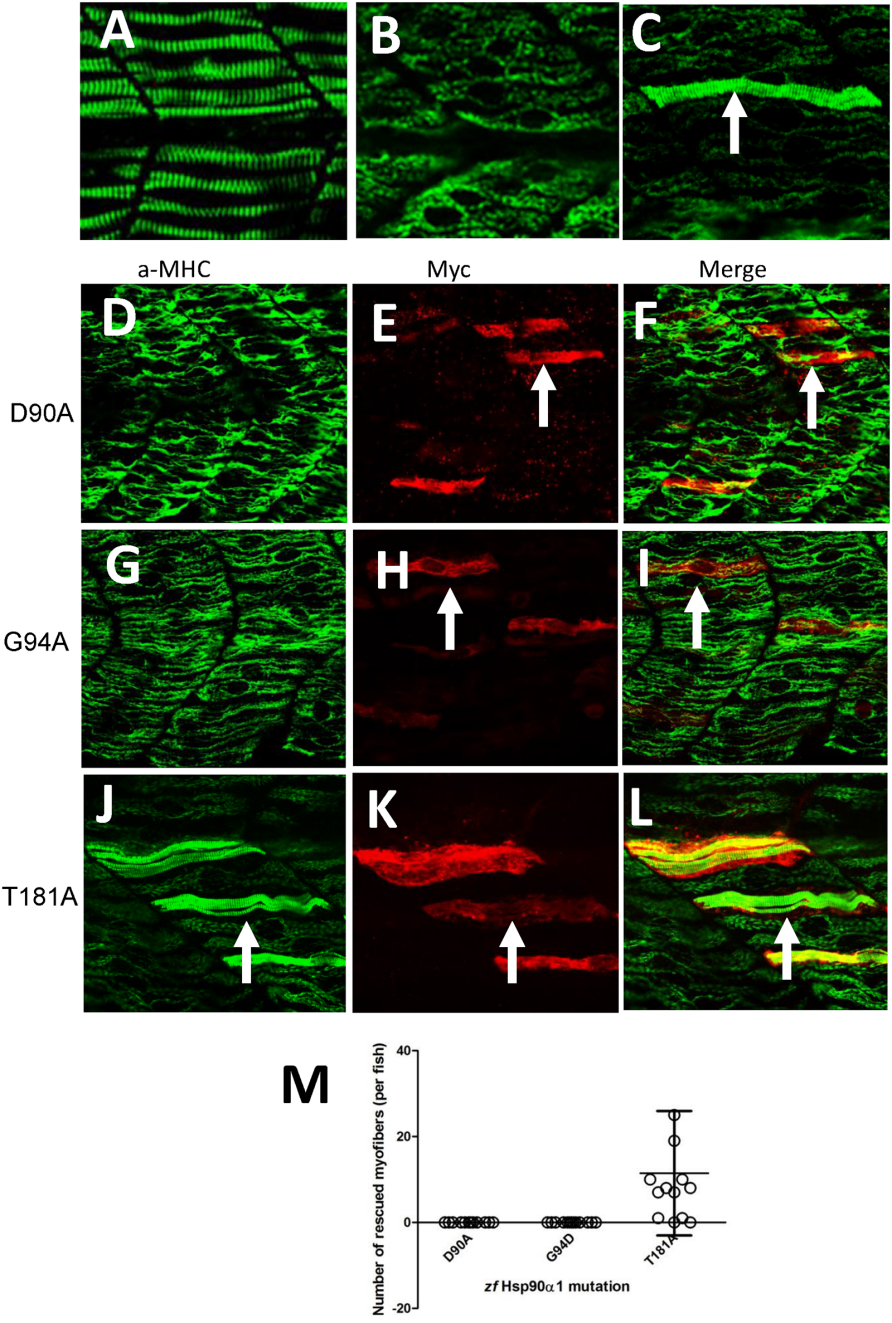
Mutating D90, G94 but not T181 in the N-terminal ATP binding domain disrupts Hsp90α1 function in myosin thick filament organization. DNA construct expressing the myc-tagged wild type Hsp90α1, D90A, G94D or T181A mutant was co-injected with Hsp90α1 ATG-MO into fertilized eggs of zebrafish. The injected embryos were analyzed by double staining with anti-myc (9E10) and anti-MHC (F59) antibodies at 28 hpf. **A-C.** Anti-MHC antibody staining shows the thick filament organization in skeletal muscles of control (A), Hsp90α1 knockdown (B), or DNA and ATG-MO co-injected (C) embryos. **D-F.** Myosin thick filament organization and myc-tagged D90A expression in skeletal slow muscles of a zebrafish embryo co-injected with Hsp90α1 ATG-MO and D90A construct. **G-I.** Myosin thick filament organization and myc-tagged G94D expression in skeletal slow muscles of a zebrafish embryo co-injected with Hsp90α1 ATG-MO and G94D construct. **J-L.** Myosin thick filament organization and myc-tagged T181A expression in skeletal slow muscles of a zebrafish embryo co-injected with Hsp90α1 ATG-MO and T181A construct. **M.** Plot showing the number of rescued myofibers in 12–13 individual zebrafish embryos injected with D90A, G94D or T184A mutant. Scale bar = 20 μm.

To determine whether D93 and T184 are critical for Hsp90α1 function in thick filament organization, their equivalent residues, D90 and T181, were identified in zebrafish Hsp90α1 and mutated to Alanine residues (Fig 1A). DNA constructs expressing the D90A or T181A mutant were analyzed in zebrafish embryos in a combined knockdown and rescue assay by co-injecting the DNA construct with the Hsp90α1 ATG-MO into zebrafish embryos (Fig 1B). Compared with the control (Fig 2A), the Hsp90α1 ATG-MO was able to knock down the expression of the endogenous Hsp90α1 gene in zebrafish embryos and resulted in defective thick filament organization (Fig 2B). However, the ATG-MO had no inhibitory effect on the expression of the *Hsp90α1* transgene because the 5’-UTR sequence targeted by the Hsp90α1 ATG-MO was removed in the transgene (Fig 1B and S1 Fig). As shown in Fig 2C, expression of a wild type *Hsp90α1* transgene was able to rescue the thick filament defect in a mosaic but cell autonomous manner.

To determine the effect of D90A or T181A mutation on Hsp90α1 function, we analyzed the thick filament organization in the co-injected zebrafish embryos by double immunostaining using anti-myosin heavy chain (a-MHC) and anti-Myc antibodies. The results showed that expression of the D90A mutant failed to rescue the myosin thick filament defects from Hsp90α1 knockdown (Fig 2D–2F). A similar muscle defect was observed in fish embryos injected with the G94D mutant that lacks the ATPase activity (Fig 2G–2I). In contrast, ectopic expression of the T181A mutant was able to rescue the thick filament defects in a cell autonomous manner (Fig 2J–2L). The result was consistent among 10–12 fish samples analyzed for each construct (Fig 2M). Together, these data indicate that D90 in the zebrafish Hsp90α1 is required for Hsp90α1 function in myofibril organization whereas T181 is not critical for Hsp90α1 function in skeletal muscles.

### 2. The effects of mutating the conserved putative phosphorylation sites on Hsp90α1 function

It has been reported that the ATPase activity is regulated by phosphorylation in yeast and human Hsp90 protein [21, 22]. Several conserved phosphorylatable sites, T22/T36, Y24/Y38 and T90, have been identified in the N-terminal domain of yeast and human Hsp90 protein (Fig 1A). Phosphorylation of T22 or Y24 residues in yeast Hsp90 or their equivalent T36 and Y38 residues in human Hsp90α reduced their ATPase activity [21, 22]. These conserved putative phosphorylation sites have been identified as T33, Y35 and T87 in zebrafish Hsp90α1. However, their regulatory role in Hsp90α1 function are not known.

**Fig 3:**
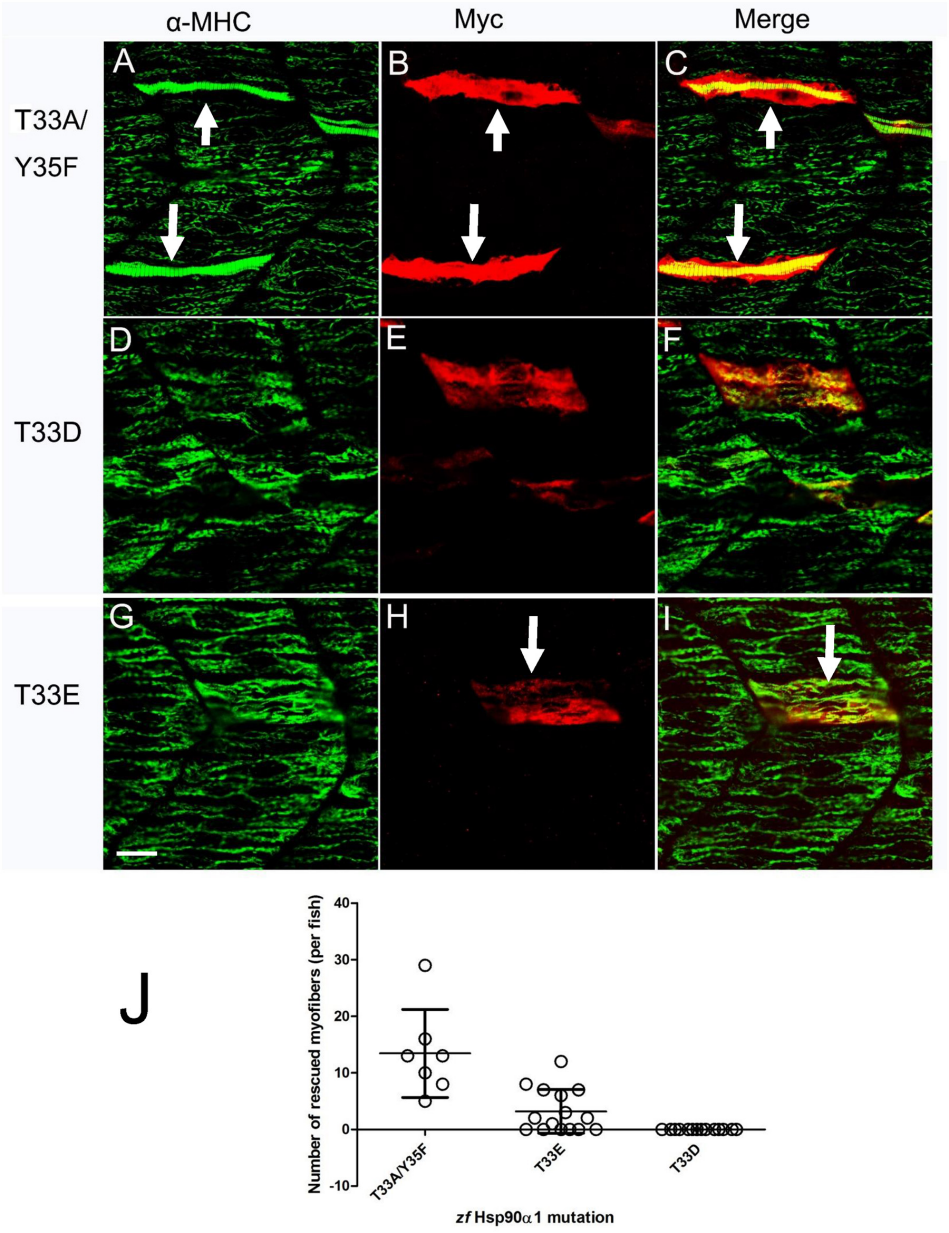
Thr33 phosphomimetic mutation blocks Hsp90α1 function in thick filament organization. DNA construct expressing the myc-tagged T33A/Y35F double, or T33E and T33D single mutant was co-injected with Hsp90α1 ATG-MO into fertilized eggs of zebrafish. The injected embryos were stained with anti-myc (9E10) and anti-MHC (F59) antibodies at 28 hpf. **A-C.** A mosaic and cell-autonomous pattern of rescue was detected in myofibers expressing the non-phosporylatable T33A/Y35F mutant. **D-I.** Expression of the phosphomimetic T33D (D-F) or T33E (G-I) mutant failed to rescue the myosin thick filament defect in skeletal muscles. **H.** Plot showing the number of rescued myofibers in 10–16 individual embryos injected with T33A/Y35F, T33E or T33D mutant. A few fibers showed a partial rescue for T33E. Scale bar = 20 μm.

To determine whether mutating these conserved putative phosphorylation sites affects Hsp90α function in myosin thick filament organization, T33, Y35 or T87 in zebrafish Hsp90α1 were mutated to either non-phosphorylatable residues, alanine (A), phenyalanine (F), or phosphomimetic residues, aspartic acid (D) and glutamic acid (E) (Fig 1A). The mutant constructs were analyzed in Hsp90α1 knockdown zebrafish embryos. The results showed that expression of the non-phosphorylatable T33A/Y35F double mutant (Fig 3A–3C) was able to rescue the myosin thick filament defect in a cell autonomous manner. In contrast, mutating T33 to a phosphomimetic residue, aspartic acid (D) or glutamic acid (E), completely blocked Hsp90α1 function in thick filament organization (Fig 3D–3I), suggesting that phosphorylation mimicking mutations at T33 inhibit Hsp90α1 function in thick filament organization in muscle cells.

**Fig 4:**
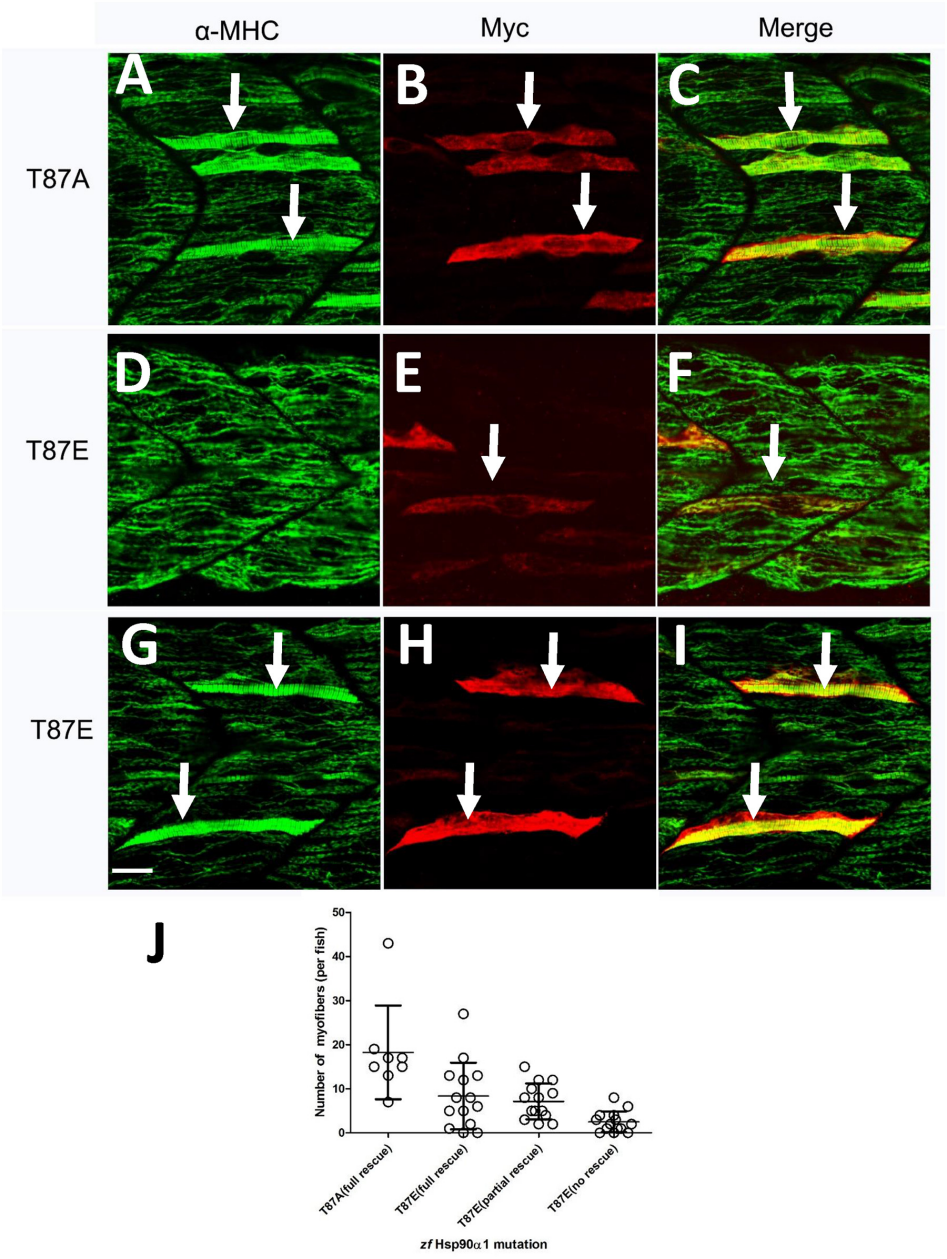
The varied effect of T87 mutation on Hsp90α1 function in myofibril organization. DNA construct expressing the non-phosporylatable T87A mutant or phospho-mimic T87E mutant was co-injected with Hsp90α1 ATG-MO into fertilized eggs of zebrafish. The injected embryos were double stained with anti-myc (9E10) and anti-MHC (F59) antibodies at 28 hpf. **A-C.** A mosaic and cell autonomous pattern of rescue was detected in all myofibers expressing the non-phosporylatable T87A mutant. **D-I.** Expression of the phospho-mimic T87E mutant resulted in a varied rescue on myosin thick filament organization. 14% (n = 35) of the T87E expressing myofibers failed to show any sign of rescue in myosin thick filament organization (D-F). In contrast, 39.5% (n = 100) and 46.5% (n = 117) of the T87E expressing slow myofibers showed a partial (not shown) or a full (G-I) rescue, respectively. J. Plot showing the number of fully rescued myofibers in T87A injected individual embryos. In addition, the numbers of fully, partially or no rescued myofibers were presented in zebrafish embryos co-injected with the T87E mutant. Scale bar = 20 μm.

Similar to T33A/Y35F mutation, our data showed that expression of the non-phosphorylatable T87A mutant was able to rescue the myosin thick filament defect in a cell autonomous manner (Fig 4A–4C). However, expression of the phosphomimetic T87E mutant showed a varied results in the rescue activity. While 14% (n = 35) of the T87E expressing fibers showed no rescue (Fig 4D–4F), 39.5% (n = 100) and 46.5% (n = 117) of the T87E expressing fibers showed a partial (data not shown) or a full rescue (Fig 4G–4I), respectively. Together, these data indicate that T87E phosphomimetic mutation had some negative impact on Hsp90α1 chaperone activity in myosin thick filament assembly. Collectively, these data indicate that similar to T33D or T33E phosphorylation mimicking mutations, T87E phosphomimetic mutation had an inhibitory role on Hsp90α1 function in myosin thick filament organization.

### 3. Mutating the conserved K287 putative acetylation site affects Hsp90α1 function in thick filament organization

**Fig 5:**
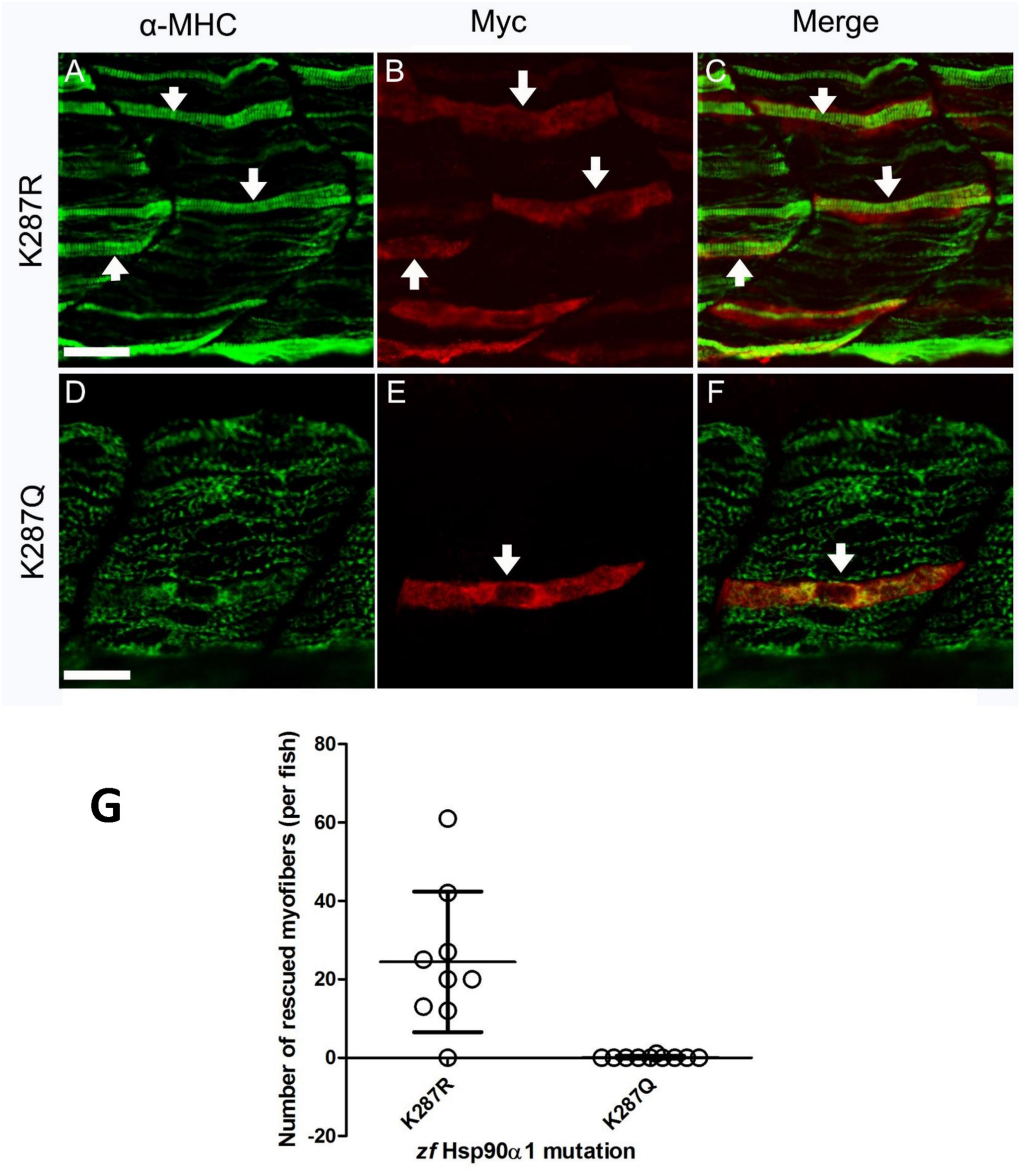
K287Q acetylation mimicking mutation affects Hsp90α1 function in thick filament organization. DNA constructs expressing the myc-tagged K287R or K287Q mutant were co-injected with Hsp90α1 ATP-MO into fertilized eggs of zebrafish. The injected embryos were stained with anti-myc (9E10) and anti-MHC (F59) antibodies at 28 hpf. **A-C.** A mosaic pattern of rescue was detected in myofibers expressing the Hsp90α1 K287R mutαnt, mimicking the unacetylated lysine. **D-F.** K287Q mutant, mimicking the acetylated lysine state, failed to rescue the myofibril defect (D-F). **G.** Plot showing the number of rescued myofibers in 8–10 individual embryos injected with K287R, or K287Q mutant. Scale bar = 20 μm.

It has been suggested that Hsp90α middle domain is involved in client protein interaction. Acetylation of K294 in the Hsp90α middle domain strongly influences mouse Hsp90 interaction with client proteins and its chaperone activity *in vitro* [14]. Sequence analysis identified the conserved K287 residue in zebrafish Hsp90α1 as the K294 equivalent in mouse Hsp90α. To determine whether mutating the K287 putative acetylation site affects Hsp90α1 function in thick filament organization, we generated two mutant constructs by replacing the K287 with either arginine (R) or glutamine (Q), mimicking the unacetylated (R) or acetylated (Q) lysine state, respectively (Fig 1A). DNA constructs expressing the K287R or K287Q mutant were co-injected with the Hsp90α1 ATG-MO into zebrafish embryos. Immunostaining showed that expression of the K287R mutant, mimicking the unacetylated Hsp90α1, was able to rescue the thick filament defects in skeletal muscles of Hsp90α1 knockdown embryos (Fig 5A–5C). In contrast, expression of the acetylation mimic mutant, K287Q, failed to rescue the myofibril defect (Fig 5D–5F). A consistent result was observed among 8–10 fish embryos analyzed for each group (Fig 5G). Together, these studies indicate that Hsp90α1 function in myosin thick filament organization could be repressed by putative acetylation of the conserved K287 residue.

### 4. Hypomethylation mimicking mutation at K206 or K608 had no effect on Hsp90α1 function in myosin thick filament organization

Proteomic analysis revealed that human Hsp90α1 is methylated at the K209 and K615 residues by Smyd2 lysine methyltransferase [23]. Knockdown of Smyd2 significantly reduced the Hsp90α methylation and resulted in defective titin organization in skeletal and cardiac muscles of zebrafish embryos [24, 25]. However, the importance of these lysine methylation on Hsp90α1 function in myosin thick filament organization has not been directly tested.

**Fig 6:**
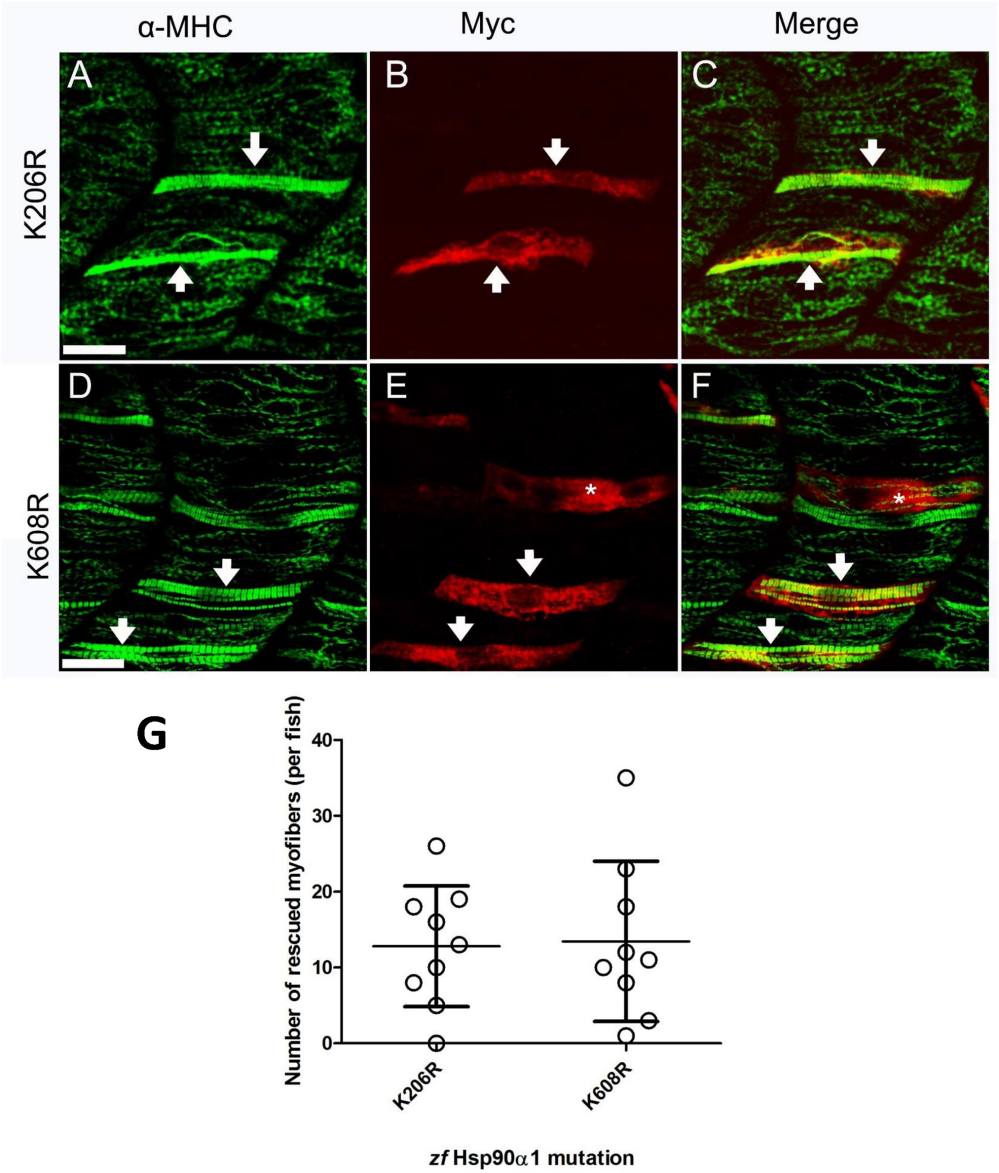
Hypomethylation mimicking mutations at K206 and K608 have no effect on Hsp90α1 function in myosin thick filament organization. DNA construct expressing K206R or K608R mutant, mimicking the hypomethylated state of lysine residue, was co-injected with Hsp90α1 ATP-MO into fertilized eggs of zebrafish. The injected embryos were double stained with anti-myc (9E10) and anti-MHC (F59) antibodies at 28 hpf. **A-F.** A mosaic pattern of rescue was detected in all myofibers expressing the Hsp90α1 K206R (A-C) or K608R (D-F) mutant. * indicates a multinucleated fast myofiber expressing K608R mutant. The anti-MHC (F59) antibody is a slow fiber specific antibody which does not label MHC expressed in fast myofibers of zebrafish embryos. **G.** Plot showing the number of rescued myofibers in 9 individual embryos injected with K206R, or K608R mutant. Scale bar = 20 μm.

To investigate the potential role of lysine methylation in Hsp90α1 function, two conserved putative methylation sites, K206 and K608, were identified in zebrafish Hsp90α1. These two putative lysine methylation sites were mutated to a arginine residue which mimics the hypomethylated lysine side chain (Fig 1A). The K206R or K608R mutant was expressed in Hsp90α1 knockdown zebrafish embryos. The results showed that expression of the K206R or K608R mutant was able to rescue the thick filament defect in Hsp90α1 knockdown embryos (Fig 6), suggesting that K206 and K608 hypomethylation did not affect Hsp90α1 function in thick filament organization. Collectively, these data argue against a potential role of K206 and K608 methylation in regulating Hsp90α1 function in thick filament organization.

## Discussion

In this study, we have developed a combined Hsp90α1 knockdown and rescue assay to characterize the structure and function of Hsp90α1 in muscle cells of zebrafish embryos. We showed that T33D and T33E phosphorylation mimicking mutations or K287Q acetylation mimicking mutation completely abolished Hsp90α1 function in thick filament organization *in vivo*. In contrast, mutations mimicking K206 and K608 hypomethylation had no effect. Given that T33 and K287 are conserved residues involved in regulation of Hsp90 function by phosphorylation and acetylation in yeast, mouse and human Hsp90 proteins, data from this study suggest that post-translational modifications by phosphorylation and acetylation may play an important regulatory role in modulating Hsp90α1 function in myosin thick filament organization.

### D90 in the ATP binding site is critical for Hsp90 function

It has been shown that ATP binding is critical for the chaperone function of Hsp90. Several key residues, D93, G97, and T184, have been identified in the ATP binding pocket of human Hsp90α that form water mediated hydrogen bond with the N1 atom of the adenine ring of an ATP molecule [18]. Functional analyses demonstrated that G97 in human Hsp90α is critical for Hsp90α ATPase activity *in vitro*, and the G94 in zebrafish Hsp90α1 is essential for Hsp90α1 function in myofibril organization in skeletal muscles of zebrafish *in vivo* [3]. Zebrafish mutant carrying a G94D missense mutation showed defective sarcomere assembly in zebrafish skeletal muscles [3].

In this study, we analyzed the role of two other key residues, D90 and T181, within the ATP binding domain for zebrafish Hsp90α1 function in myosin thick filament organization. Consistent with the structural data, we demonstrated that D90A mutation abolished its biological function in myofibril organization. In contrast, T181A mutation had no effect on Hsp90α1 function in thick filament organization. Collectively, these data support a role for D90 in zebrafish Hsp90α1 function and argue against the critical role of T181 in Hsp90α1 function.

### Regulation of Hsp90α1 function by putative phosphorylation

Our studies demonstrated that phosphorylation mimicking mutations of T33D or T33E abolished Hsp90α1 function in myosin thick filament organization. Our functional data from zebrafish muscles are consistent with previous biochemical studies showing that the Hsp90 ATPase activity is controlled by phosphorylation in yeast and human [21, 22]. It has been shown that phosphorylation mimicking mutations of yeast T22E or human T36E markedly decreased the Hsp90 ATPase activity *in vitro* [21, 22]. Given that T33A mutation of zebrafish Hsp90α1 had no effect on its function in myofibril organization whereas the phosphorylation mimic mutation T33D completely abolished Hsp90α1 function in thick filament organization, these data argue a potential inhibitory effect of Hsp90 function by T33 phosphorylation. Mollapour and colleagues [22] have reported that casein kinase 2 phosphorylates yeast Hsp90 at the Thr22 residue, it remains to be determined whether T33 in zebrafish Hsp90α1 is indeed methylated and whether the methylation is mediated by casein kinase 2 in skeletal muscle cells.

The molecular mechanism by which phosphorylation regulates Hsp90 ATPase activity and function is not clear. It has been shown that the conserved threonine residue identified in the N-terminal α helix-1 of yeast (T22) and human (T36) Hsp90 is involved in a hydrophobic interaction with the catalytic loop in the Hsp90’s middle domain. The α helix-1 in Hsp90α1 is required for the stabilization of the chaperone’s ATPase-competent state [22]. Phosphorylation of T22/T33/T36 may affect the α helix-1 structure of Hsp90 and its interaction with the catalytic loop of the ATPase domain. Consistent with the idea that phosphorylation of Hsp90 may weaken its ATPase activity, overexpression of Aha1, the activator of Hsp90 ATPase, is able to compensate for the reduced chaperone activity of the Hsp90α phosphomimetic mutants [22].

### Regulation of Hsp90α1 function by putative acetylation

We showed in this study that the conserved K287 residue implicated in acetylation is critical for Hsp90α1 function in thick filament organization. K287Q mutation, mimicking acetylated lysine, completely abolished Hsp90α1 function in thick filament organization. Our data are consistent with a previous report that K294Q mutation which mimics the acetylation state significantly abrogated the Hsp90 chaperone activity and binding with client proteins and cochaperones *in vitro* [14].

The molecular mechanism by which acetylation regulates Hsp90α1 function is not clear. It has been reported that K294 acetylation does not affect ATP binding of yeast Hsp90α [14]. Acetylation may control Hsp90α function via modulating its interaction with cochaperones and client proteins. Structural analyses revealed that K294 is located at the beginning of the Hsp90 middle domain involved in both intramolecular contacts as well as dynamic protein-protein interactions with both client proteins and cochaperones [26, 27]. It has been reported that Aha1 binds to the middle domain of Hsp90, contributes to client protein activation, and stimulates the ATPase activity [28]. It has been reported that acetylation mimicking mutation (K249Q) significantly attenuated Hsp90α interaction with Aha1, whereas K294R mutation which mimics the unacetylated state displayed a normal or even stronger interaction with Aha1 [14]. This is consistent with our finding that K287R mutation, mimicking the unacetylated lysine, had no effect on Hsp90α1 function in thick filament organization, whereas the acetylation mimicking mutation abolished Hsp90α1 function.

### Regulation of Hsp90α1 function by methylation

It has been suggested that lysine methylation regulates Hsp90α activity in sarcomere assembly [24, 25]. Proteomic analysis revealed that Smyd2 methylates Hsp90α at K209 and K615 lysine residues [23]. Knockdown of Smyd2 resulted in the loss of Hsp90α methylation, impaired titin stability, and altered muscle function in skeletal and cardiac muscles [24, 25]. However, it is not clear whether loss of Hsp90α1 methylation at K209 and K615 caused the muscle defects. We demonstrated in this study that mutating the two putative lysine methylation residues to a hypomethylated state had no effect on Hsp90α1 function in thick filament organization. The Smyd2 knockdown phenotype could not be phenocopied by the loss of Hsp90α1 methylation, suggesting that Smyd2 may have other function in addition to Hsp90α1 methylation in myofibril organization. However, given that Hsp90 has a large numbers of client proteins, we could not rule out the possibility that the K209 and K615 hypomethylation mimicking mutations may affect the function of Hsp90 on other sarcomere proteins, such as titin [24, 25].

In summary, we have developed a combined knockdown and rescue assay in zebrafish embryos to study the potential regulation of Hsp90α1 function by putative post-translational modification. Our studies revealed that Hsp90α1 mutations mimicking phosphorylation or acetylation abolished Hsp90α1 function in myofibril assembly. However, it should be noted that we have no direct evidence that these residues are modified by various PTMs in zebrafish Hsp90α1 although these conserved residues subjected to phosphorylation and acetylation have already been reported in human and yeast Hsp90 proteins. It has been increasingly recognized that post-translational modification of Hsp90 is a complex and dynamic process involving phosphorylation, acetylation and methylation [10, 11]. The various combination of potential Hsp90 PTMs could provide a regulatory mechanism for repression or activation of Hsp90α1 function in muscle cells.

## Materials and Methods

### Ethics statement

This study was carried out according to the guideline for the Care and Use of Laboratory Animals of the National Institutes of Health. After the completion of the experiments, the animals were euthanized using tricaine methane sulfonate (MS 222) at a concentration of 250 mg/L. The protocol was approved by the Institutional Animal Care and Use Committee of the University of Maryland (Permit Number: 0513005).

### Zebrafish maintenance

Adult zebrafish were raised at the Aquaculture Research Center, Institute of Marine and Environmental Technology (Baltimore, MD). The fish were maintained at 28°C with a photoperiod of 14 h light and 10 h dark in 8-gallon aquaria supplied with freshwater and aeration. Fish spawning and egg collection will be carried out by natural crosses of adult fish. This procedure does not require surgery or anesthesia.

### Morpholino and DNA microinjection

Hsp90α1 translation blocker (ATG-MO) was synthesized by Gene Tools (Carvalis, OR). The ATG-MO was dissolved in Danieau buffer [29] to a final concentration of 0.5 mM. The ATG-MO (0.5 mM) was mixed with equal volume of DNA expression construct (100 ng/μl) encoding various Hsp90α1 mutants. 1–2 nl of the mixture containing 4–8 ng of ATG-MO and 50–100 pg of DNA construct was injected into each embryo at one or two cell stage. The standard control oligo from Gene Tools was used as the negative control.

Hsp90α1 ATG-MO: 5’ CGACTTCTCAGGCATCTTGCTGTGT- 3’

### Construction of *Tg(smyd1*:*Hsp90α1* ^*myc*^ *)* mutant gene constructs

DNA constructs expressing various Hsp90α1 mutations were generated using a QuikChange^®^ site-directed mutagenesis kit (Stratagene). The *Tg(smyd1*:*Hsp90α1* ^*myc*^) construct expressing a myc-tagged wild type zebrafish Hsp90α1 was used as the template for the mutagenesis [2]. The zebrafish *smyd1* muscle specific promoter was used to drive the expression of wild type or mutant Hsp90α1 in muscle cells of zebrafish embryos [30]. The various Hsp90α1 mutant constructs and PCR primers used in the mutagenesis were shown in S1 Table. All DNA expression constructs were confirmed by DNA sequencing.

### Analysis of protein expression by Western blot in injected zebrafish embryos

Expression of myc-tagged wild type or mutant Hsp90α1 proteins was determined by western blot analysis in the injected embryos. Western blot analysis was performed using the anti-myc antibody 9E10 (Developmental Studies Hybridoma Bank, DSHB) as described [2, 31]. Briefly, zebrafish embryos were manually dechorionated at 22 hpf using forceps. Yolk sacs of fish embryos were removed by gently pipetting through a glass pipet in 1 ml of PBS buffer. The deyolked embryos were collected by low speed centrifugation at 3000 rpm for 1 min. Total proteins of fish embryos were solubilized in 2×SDS lysis buffer (3 μl for each embryo) containing 1mM PMSF and protease inhibitors (P8340, Sigma-Aldrich). 30 μl of protein sample (10 embryos) was loaded each lane on a 10% SDS-PAGE. Immunoblot was performed using anti-Myc (9E10; DSHB), and anti-γ-tubulin (T6557, Sigma) antibodies, and HRP labeled anti-mouse secondary antibody (7076S, Cell signaling).

### Whole-mount double immunostaining

The whole mount immunostaining was carried out with the injected embryos at 28 hpf using the F59 anti-myosin heavy chain antibody (DSHB), and anti-Myc antibody (#2278s, Cell Signaling) as previously described [2]. Anti-mouse IgG and anti-rabbit IgG secondary antibodies were labeled with Alexa Fluor^®^ 488 Dye (A31619, Invitrogen) or Alexa Fluor 555 dye (A31630, Invitrogen), respectively.

### Statistical analysis

Statistical analyses were conducted using the statistical software GraphPad Prism 5.0 (GraphPad, La Jolla, California, USA). The number of rescued fiber was shown as pilot and Mean±SD.

## Supporting Information

S1 Fig Western blot analysis showing the expression of myc-tagged wild type and mutant Hsp90α1 proteins in zebrafish embryos co-injected with Hsp90α1 ATG-MO. (PDF)

S1 Table List of PCR primers used in mutagenesis. (PDF)

## Abbreviations

D90: Aspartic acid 90
Hsp90α: Heat shock protein 90α
Hpf: hour-post-fertilization
K208, K287 and K608: Lysine 206, Lysine 287 and Lysine 608
MO: Morpholino
T33 and T87: Threonine 33, Threonine 87
