# Supplementary material for: The Effects of Hsp90α1 Mutations on Myosin Thick Filament Organization
Source: PLoS One. 2015 Nov 12;10(11):e0142573. doi: 10.1371/journal.pone.0142573 (PMC4642942; doi:10.1371/journal.pone.0142573)
Supplement: S1 Fig — (PDF) [file pone.0142573.s001.pdf]

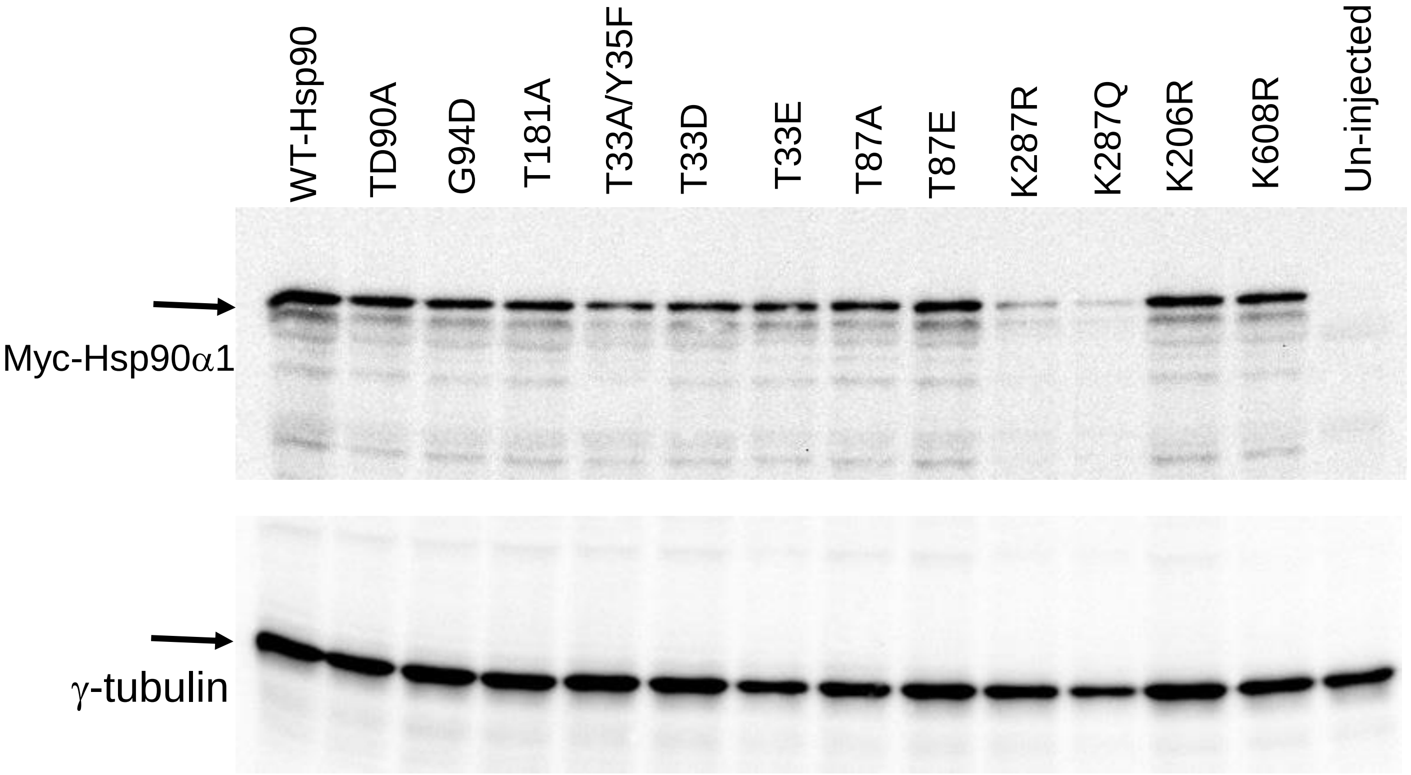

S1 Figure. Western blot analysis showing the expression of myc-tagged wild type and mutant Hsp90α1 proteins in zebrafish embryos co-injected with Hsp90α1 ATG-MO. Expression of the myc-tagged proteins was analyzed by Western blot using anti-myc tag antibody (9E10). Each lane contains protein extract from 10 embryos. γ-tublin was used as a loading control.
