# Supplementary material for: The Effects of Hsp90α1 Mutations on Myosin Thick Filament Organization
Source: PLoS One. 2015 Nov 12;10(11):e0142573. doi: 10.1371/journal.pone.0142573 (PMC4642942; doi:10.1371/journal.pone.0142573)
Supplement: S1 Table — (PDF) [file pone.0142573.s002.pdf]

Supporting information

S1 Table. List of PCR primers used in mutagenesis

Site mutation	Mutation primer sequence
T33A/Y35F	P1: CTCTGATCATCAACGCCTTCTTCTCCAACAAGGAGA<br>P2: TCTCCTTGTTGGAGAAGAAGGCGTTGATGATCAGAG
T33D	P1: CTCTGATCATCAACGACTTCTACTCCAACAAG<br>P2: CTTGTTGGAGTAGAAGTCGTTGATGATCAGAG
T33E	P1: CTCTGATCATCAACGAGTTCTACTCCAACAAG<br>P2: CTTGTTGGAGTAGAACTCGTTGATGATCAGAG
T87A	P1: AAGAGCGCACGCTGGCCATCATCGACACC<br>P2: GGTGTCGATGATGGCCAGCGTGCGCTCTT
T87E	P1: AAGAGCGCACGCTGGAGATCATCGACACCGG<br>P2: CCGGTGTCGATGATCTCCAGCGTGCGCTCTT
D90A	P1: CGCTGACCATCATCGCCACCGGCATCGGCAT<br>P2: ATGCCGATGCCGGTGGCGATGATGGTCAGCG
G94D	P1: TCGACACCGGCATCGACATGACCAAAGCTGA<br>P2: TCAGCTTTGGTCATGTCGATGCCGGTGTCGA
T181A	P1: ATCAATTGGACGTGGTGCCAAAGTCATTCTCCAC<br>P2: GTGGAGAATGACTTTGGCACCACGTCCAATTGAT
K206R	P1: GGAAGTGGTGAAGAGGCACTCTCAGTT<br>P2: AACTGAGAGTGCCTCTTCACCACTTCC
K287R	P1: GCTGAATAAGACCAGGCCGATCTGGACC<br>P2: GGTCCAGATCGGCCTGGTCTTATTCAGC
K287Q	P1: GCTGAATAAGACCCAGCCGATCTGGACC<br>P2: GGTCCAGATCGGCTGGGTCTTATTCAGC
K608R	P1: GGAGAGGATCATGAGGTCTCAGGCTCT<br>P2: AGAGCCTGAGACCTCATGATCCTCTCC
